# Editorial: Trajectories of Brain Abnormalities in Early Schizophrenia

**DOI:** 10.3389/fpsyt.2021.744471

**Published:** 2021-08-31

**Authors:** Antonio Vita, Luca De Peri, Stefan Borgwardt

**Affiliations:** ^1^Department of Mental Health, Spedali Civili Hospital, University of Brescia School of Medicine, Brescia, Italy; ^2^Cantonal Psychiatric Clinic, Cantonal Socio-Psychiatric Association, Mendrisio, Switzerland; ^3^Department of Psychiatry, University of Basel, Basel, Switzerland; ^4^Department of Psychiatry, Psychosomatics and Psychotherapy, University of Lübeck, Lübeck, Germany

**Keywords:** early schizophrenia, structural brain imaging, functional brain imaging, pathophysiological trajectory of brain abnormalities, outcome

Several brain abnormalities in schizophrenia have been demonstrated by a large number of structural and functional neuroimaging studies. The nature and meaning of such abnormalities, their time of occurrence, whether they are static or progressive as well as the role of potential confounders of brain changes have been investigated by several studies over the last 40 years. The findings of these investigations support the notion that some structural and functional brain anomalies predate the onset of schizophrenia and are detectable to a larger extent in first-episode patients (FEP). Despite the large amount of literature on brain changes in the early stages of schizophrenia, the findings to date do not allow definite conclusions to be drawn about the pathophysiological trajectory of schizophrenia and to resolve the dispute between the so-called “neurodevelopmental” and “neurodegenerative” hypotheses of brain abnormalities of the disorder. The papers included in this Research Topic on “*Trajectories of Brain Abnormalities in Early Schizophrenia”* provide some new insights to address the question of which or how many trajectories of brain changes can be detected in the early phases of the disorder and to which extent they are related to the multidimensional outcome of the disorder.

Here we present a brief overview of the contents and highlights of the present Research Topic. A number of original papers investigated clinical and biological predictors of outcome in first-episode schizophrenia (FES). Takahashi et al. analyzed longitudinal gray matter changes in the insular cortex of FES patients during a 3 years Magnetic Resonance Imagine (MRI) follow-up evidencing dynamic volumetric changes at different time points over the early course of illness. Kim et al. investigated throughout diffusion tensor imaging (DTI) the association between white matter integrity and Theory of Mind (ToM) impairment in first-episode patients compared to healthy controls. The study findings suggested a possible neural basis for ToM deficits in schizophrenia. Moreover, In their contribution, González-Vivas et al. analyzed longitudinal changes in brain fMRI BOLD over a 2 years follow-up in first-episode patients and healthy controls. Interestingly, the study also took into account the potential moderating role of antipsychotic medication on brain functional measurements. Cheng et al. investigated the clinical predictors of persistent remission (PR) within the 1st year of antipsychotic treatment in a large sample of first-episode patients (*n* = 301). Among several psychopathological and neurocognitive variables considered, the results of the study showed that the severity of negative symptoms at baseline was the best predictor of PR within 1 year of antipsychotic treatment.

Two original studies in this issue investigated structural and functional brain parameters in patients at clinically (CHR) or genetically (GHR) high risk for psychosis. Takayanagi et al. performed a cross-sectional structural MRI study of the planum temporale (PT) comparing 73 individuals with an at-risk mental state (ARMS) and 74 healthy controls using labeled cortical distance mapping. The study did not show significant between groups differences of PT measures, suggesting that structural changes associated with schizophrenia may occur just before or during the onset of the disorder. Collin et al. examined the default mode network (DMN) in children at familial high risk (FHR) for psychosis compared to healthy children. The study evidenced an hyperactivity of posterior DMN in pre-adolescent FHR children as compared to healthy controls. As suggested by the authors, this finding may posit DMN-related disturbances as a developmental brain abnormality associated with familial risk factors that predates psychosis.

Two electrophysiological studies in early schizophrenia also contributed to this special issue. Mackintosh et al. investigated the effect of antipsychotic medication on EEG parameters in FEP patients. The results demonstrated significant differences of EEG resting-state microstates between medicated and non-medicated FEP patients. Mazer et al. studied auditory event-related potentials (ERP) in a cross-sectional controlled study. In particular, the paper examined the modulation and habituation of ERPs early components N1 and P2 in 26 FES patients and 27 healthy participants. The study findings evidenced anomalies of sensory gating processes yet in the course of early schizophrenia.

Finally, some qualitative and quantitative reviews completed the present Research Topic, with an useful update on biological research in early schizophrenia. In their contribution, Kraguljac and Lathi critically considered a number of key scientific questions both methodologically and clinically relevant in the context of neuroimaging studies to understand the pathophysiology of schizophrenia. Yang et al. performed a selective review of 36 structural and functional longitudinal MRI studies in FES patients before and after antipsychotic treatment. Kose et al. conducted a qualitative review of the evidence regarding the potential role of peripheral inflammatory markers as predictors of treatment response in schizophrenia and the association between peripheral inflammatory markers with neuroimaging data. Merritt et al. reported a qualitative review to determine longitudinal structural MRI findings in GHR and CHR individuals for psychosis. Lastly, Hinney et al. investigated, through a meta-analytical approach, whether hippocampal volume may serve as a biomarker for psychosis. This quantitative review included those studies that compared hippocampal volume changes over time in patients at risk for psychosis who developed illness as compared to patients who do not have such a transition.

In conclusion, the present collection of research papers offers a multidisciplinary perspective of the topic of trajectories of brain abnormalities in early schizophrenia, which may help to understand the pathophysiology of the disorders and contributes to the discussion on the etiopathogenetic hypotheses of the disorder. The main implications provided from the studies collected in the topic can be summarized as follows: (1) there is evidence of dynamic structural and functional brain changes at different time points in the early course of schizophrenia; (2) brain changes in early schizophrenia involves both gray and white matter structures; (3) structural and functional brain anomalies are detectable even before the overt clinical onset of the disease as demonstrated by longitudinal studies in patients clinically or genetically at risk for schizophrenia; (4) antipsychotic medication represents a major confounder of brain changes over time in schizophrenia. This evidence once again questions the clinicians about the risk/benefit balance of antipsychotic treatment also in the early course of the disease.

With all these clinical and research implications in mind, we wish that the present Research Topic may receive interest and activate debate among the readers and contributors and solicit new ideas for original research and further hypotheses on this fascinating field of psychiatric research. The future directions of neuroimaging research in schizophrenia indicate the need to set new methodological standards both for developing truly innovative hypothesis-driven neuroimaging research and for reliably interpreting structural and functional multimodal imaging databases with the aim to overcome the current limits and to reach the target of diagnostic and prognostic biomarkers at the individual subject level. Otherwise, the scientific community will have to deal with redundant or weak findings with the potential risk of dissipating even well-grounded notions about the pathophysiology of schizophrenia deriving from almost 50 years of neuroimaging research.

## Author Contributions

AV and LDP wrote the paper. SB revised and completed the Editorial. All authors contributed to the article and approved the submitted version.

## Conflict of Interest

The authors declare that the research was conducted in the absence of any commercial or financial relationships that could be construed as a potential conflict of interest.

## Publisher's Note

All claims expressed in this article are solely those of the authors and do not necessarily represent those of their affiliated organizations, or those of the publisher, the editors and the reviewers. Any product that may be evaluated in this article, or claim that may be made by its manufacturer, is not guaranteed or endorsed by the publisher.

